# Influence of Cooling Rate on the Flexural and Impact Properties of Compression Molded Non-Woven Flax/PLA Biocomposites

**DOI:** 10.3390/polym17040493

**Published:** 2025-02-13

**Authors:** Anurag Pisupati, Marco Curto, Thomas Laurent, Benoit Cosson, Chung Hae Park, Hom Nath Dhakal

**Affiliations:** 1 Center for Materials and Processes, IMT Nord Europe, Institut Mines-Télécom, Université de Lille, 59000 Lille, France; anurag.pisupati@ct-ipc.com (A.P.); benoit.cosson@uttop.fr (B.C.); 2 Wärtsilä Defence Solutions UK Limited, 4 Marples Way, Havant PO9 1NX, UK; marco.curto@wartsiladefence.com; 3 Centre Européen des Textiles Innovants, 59355 Tourcoing, France; t.laurent@team2.fr; 4 Advanced Polymers and Composites (APC) Research Group, School of Mechanical and Design Engineering, University of Portsmouth, Portsmouth PO1 3DJ, UK

**Keywords:** flax fiber, polylactic acid (PLA), crystallinity, flexural properties, process cycle time, thermal degradation

## Abstract

This work investigates the influence of crystallinity on the mechanical properties of needle-punched non-woven flax/polylactic acid (PLA) biocomposites with different flax fiber contents. Biocomposites were fabricated by a compression molding adopting different cooling rates to understand the mechanism of crystallinity and their contribution to the mechanical properties. Image-based analysis of the fiber distribution in non-woven preform indicates the probable origins of the residual porosities and the potential nucleation sites for crystal formation within the composites. The improvement of 25% and 100% in flexural modulus is observed for the composites with 40% and 50% of flax fiber mass fractions, respectively, when subjected to a lower cooling rate, which implies the significant influence of the void content on the brittleness of composites. The impact properties of the composites decrease from 11% to 18% according to the flax fiber mass fraction when the cooling rate decreases to 1 °C/min, and the composites become more brittle. The induced impact and flexural properties of the composites are compared with those of other composites in the literature to emphasize their applicability to semi-structural applications.

## 1. Introduction

Flax fibers as composite reinforcement are of great interest owing to their reasonable cost, low density, and high specific mechanical properties, and they are a promising replacement for glass fibers in composite materials. In many industrial parts, however, the applicability of flax fiber thermoplastic composites is limited to semi-structural applications or non-structural applications. These composites are manufactured using woven or non-woven (oriented or random) fabrics and mats. Although woven reinforcements are of great interest owing to high mechanical performance and drapability, their cost is also relatively higher than their counterparts, i.e., non-wovens. The non-woven fabric reinforcements are becoming popular in the automotive industry for semi-structural or non-structural components since the product cost becomes lower than that of woven fabric parts, which is one of the key driving factors in the automotive sector. Moreover, these non-woven fabrics are easy to handle and provide greater formability. Additionally, the non-wovens with random fiber arrangement provide quasi-isotropic behavior, unlike oriented reinforcements [1], which can be tailored according to the required loading direction and the application. Furthermore, these random non-woven composites exhibit mechanical performance higher than the injection-molded composites [2] and can be molded into large complex shapes without drapability issues.

Most thermoplastic flax composites adopt polyolefins matrices [3,4,5,6,7]. Using thermoplastics such as polylactic acid (PLA) or other bio-based thermoplastics can be interesting, owing to their easier end-of-life treatment [3,5,8]. Several studies have investigated the feasibility of flax/PLA composites in the past two decades [9,10,11,12,13,14,15,16,17,18]. Most of these studies employed injection or compression molding to manufacture flax/PLA composites. The limitation of injection molding is that one cannot achieve high fiber volume fractions (*V_f_*), whereas, with compression molding, high fiber volume fractions such as over 30% can easily be achieved. Furthermore, the fiber length can be much shorter in the case of injection molded composites leading to low mechanical performance.

Compression molding is one of the main manufacturing processes used to produce woven and non-woven polymeric composite parts. Compression molding of composites can be sub-categorized into several manufacturing routes depending on the state of reinforcements. In the case of prepregs, the process is simple and straightforward: the prepregs are cut to the mold shape and consolidated under pressure and heat [19]. In the case of film stacking, the influence of through-thickness permeability comes into play, which significantly affects the impregnation quality [20,21]. A higher processing temperature can be imposed to avoid poor impregnation issues and achieve composite parts with low void content. Temperatures higher than 200 °C are not advised for flax fibers, however, because they may lead to the thermal degradation of flax fibers [7]. Another solution is using commingled fabrics since they reduce the resin flow path and process cycle time, which is a prerequisite for high production volume manufacturing. Commingled fabrics can also be transformed into tapes, which can be used in laser-assisted tape placement for manufacturing complex-shaped parts [22,23] or can be directly used for other manufacturing processes such as pultrusion [24,25,26] and additive manufacturing [27]. Commingled fabrics can be in the form of fabrics with hybrid yarns or needle-punched non-wovens. In the case of hybrid yarns, thermoplastic and natural fiber yarns are twisted to maintain the form and facilitate weaving, but this leads to poor impregnation and low mechanical performance. As the natural fiber yarns are twisted, the resin penetration can be difficult, leading to high void content. This arrangement also leads to a significant drop in the mechanical properties of the composites [28,29,30,31].

Polylactic acid (PLA) is a semi-crystalline polymer whose properties are highly sensitive to the cooling rate imposed during composite processing. Rapid cooling results in reduced crystallinity by limiting the time available for crystal growth at nucleation sites, such as flax fiber surfaces, whereas slower cooling promotes crystallization, allowing for the development of more ordered crystal structures [32,33]. Although rapid cooling is often favored to decrease production time, it can detriment mechanical properties if crystallization is insufficient. This relationship between cooling rate and mechanical performance has been well characterized for glass fiber-reinforced composites [34], yet remains underexplored for flax fiber-reinforced PLA composites.

The relationship between the degree of crystallinity and flax fiber content in PLA composites has been sparsely studied [33,35]. While several investigations in the literature have detailed the crystallization behavior of PLA in the presence of natural fibers [36,37,38], the scope of this current work is specifically limited to flax fibers at high mass fractions. Xia et al. [33] demonstrated that increasing flax fiber content enhances the transcrystallinity of the PLA matrix, attributing this to the nucleating effect of the fibers. Similarly, Aliotta et al. [35] reported that a higher fiber content leads to increased PLA crystallinity, which improves the mechanical properties of the resulting composites. Furthermore, Bayart et al. [32] improved the crystallization and interfacial adhesion of flax/PLA composites by incorporating titanium dioxide. However, these studies lack comprehensive analyses of the interplay between the crystallization kinetics and the mechanical performance of flax/PLA composites. This study aims to bridge these gaps by systematically investigating the contribution of crystallization kinetics to the mechanical properties of non-woven flax/PLA composites with high fiber mass fractions (M_f_ > 30%). The research involves characterizing the mechanical and thermal properties of the composites and comparing the results with those in the existing literature. Furthermore, the crystallization kinetics are modeled using analytical approaches. The non-woven composites are fabricated under consistent thermal processing conditions to ensure reproducibility, and their microstructures are examined to establish correlations between the process cycle, crystallinity, and resultant mechanical properties. This comprehensive approach provides a deeper understanding of how thermal processing affects the structural integrity and applicability of flax/PLA biocomposites in semi-structural applications.

## 2. Materials and Methods

### 2.1. Reinforcement and Matrix Materials

**Figure 1:**
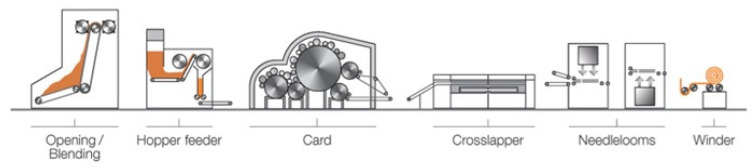
Schematic representation of the carding process.

In this study, needle-punched non-wovens were prepared using commercial PLA filaments from Max model, France, and flax fibers from Procotex SA, Mouscron, Belgium. The preparation steps of dry-laid fabrics are shown in Figure 1. These fibers were delivered in an opening Laroche system using two Laroche feeding rooms. The fibers were opened and weighed to provide an excellent blend with the expected mixture rate to a carding system. The fibers were laid down on a conveying belt and superposed to be fed to the opening system. The feeding system was composed of one pin cylinder that nibbled two layers of fibers and fed a constant and open fiber blend. The fibers were then fed to a carding machine (Excelle Andritz, Graz, Austria) composed of a pre-working unit with three carding groups, a main cylinder with five carding cylinders, and two doffers. The veils coming from these cards were then cross-lapped to form non-woven mats that were bonded through needle punching. The target areal weight of the fabrics was set to 175 g/m^2^ with a nominal thickness of 1.8 mm. Three different configurations were manufactured in this study with varying flax mass fractions. The following nomenclature was adopted for non-wovens and their composites: F-XX, where F denotes flax and XX refers to the mass fraction of flax fibers. The actual areal weight of the fabric was measured by weighing at least three large fabric samples with dimensions of 500 × 500 mm^2^. The variation in the areal weights could be caused by the variability in the carding process of both fibers. Furthermore, to investigate the spatial distribution of flax and PLA fibers within the non-woven, the optical measurements of fiber distribution were carried out according to the method suggested by Cosson [39]. This approach provides the information about the distribution of fibers and the fiber orientations within the fabric, which can then be used to predict the degree of anisotropy of the composite properties.

### 2.2. Fabrication of Composite Laminates

**Figure 2:**
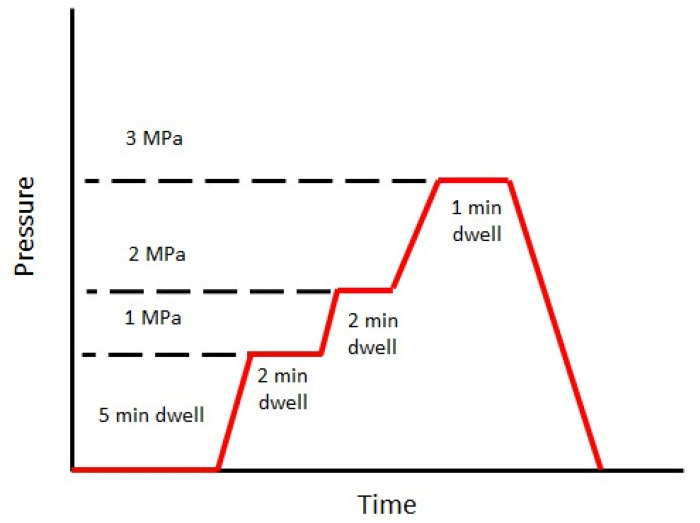
Stepped consolidation cycle in compression molding.

In this study, the composite laminates were manufactured using the compression molding technique. The non-woven fabrics were cut to the mold dimensions, i.e., 250 × 150 mm^2^, and were stored in a conditioning room (23 °C and RH 50%) for at least 24 h and were not dried before the manufacturing. Owing to the varied areal weight of fabrics (see Table 1), the number of layers in each composite plate was not maintained constant. Nevertheless, the total weight of the stack was maintained constant for each plate. The weight of the stack was calculated based on the fiber densities of flax and PLA mentioned in Section 2.1. The cavity height of the mold was 2 mm. The non-woven fabrics were stacked and transferred onto a preheated hydraulic press (PEI, Charlon sur Saône, France). For rapid cooling, the mold was immediately transferred to another hydraulic press (Douloets, Soustons, France) maintained at 23 °C. All the composites were demolded when the mold temperature reached about 40 °C. The adopted consolidation cycle is shown in Figure 2. In this study, a stepped consolidation cycle was selected based on the recommendations of a previous study [7,40]. A stepped consolidation cycle is advantageous for two reasons: since the preform is compacted sequentially, the polymer flow will induce lower drag forces on the fibers, avoiding significant movement during the consolidation step. This approach also ensures that the entrapped air moves out easily. Furthermore, if a sudden pressure is applied on a smaller surface area, there could be significant squeeze flow, which would induce defective parts. At least three plates were manufactured for each composition.

### 2.3. Flax Fiber Distribution

To understand the variability of the distribution of flax fibers, the optical approach suggested by Cosson was adopted in this study [39]. The method employs a bracketing approach that facilitates the calculation of energy received by the camera and facilitates the calculation of the mean difference to estimate the fiber distributions [41,42]. The approach is macroscopic and ensures faster analysis compared to microscopy or other advanced techniques. Single-layer preforms of dimensions 250 × 150 mm^2^ were consolidated at 170 °C and rapidly cooled to ensure no crystals were formed. These single-layer composites were placed against a light source (a 17-inch LED laptop screen) to capture the images. These images were further processed using MATLAB to report the local variations. The image treatment steps are described elsewhere [39].

### 2.4. Void Content Measurement

The density of the non-woven composites was measured using the Archimedes principle employing ethanol as the test liquid. The samples with dimensions of 20 × 20 mm^2^ were cut from the plates randomly to report the average values. Using the apparent density of composites, the void volume fraction of the composite was estimated according to the CRAG report [43]. The density of flax and PLA was assumed to be 1.5 g/cm^3^ [44] and 1.24 g/cm^3^ [40], respectively.

### 2.5. Thermal Characterization

#### 2.5.1. Differential Scanning Calorimetry (DSC)

Differential scanning calorimetry (DSC) was used to identify the crystallization kinetics of the pure polymer (i.e., PLA) and flax/PLA non-wovens. The DSC test samples were hermetically sealed in an aluminum crucible and weighed about 6–8 mg. The samples were subjected to a sequential heating and cooling cycle. The heating rate for all the tests was maintained constant at 5 °C/min. To analyze the nonisothermal crystallization, the samples were subjected to three different cooling rates, viz., 1 °C/min, 5 °C/min, and 10 °C/min. On the other hand, isothermal crystallization was investigated at three different temperatures, viz., 90 °C, 100 °C, and 120 °C. These temperature values were selected based on the literature review to analyze the different crystallization behaviors of PLA [45]. The crystallinity (*χ*) was calculated based on the melting (ΔH_m_) and cold crystallization enthalpy (ΔH_cc_) of the polymer using the following equation (Equation (1)).

(1) $$\chi =\frac{\Delta H_{m}-\Delta H_{cc}}{\left(1-\phi \right)\Delta H_{100\%PLA}}\;\times \;100$$

where Δ*H*_100%*PLA*_ corresponds to the complete melting enthalpy of PLA, equivalent to 93.2 J/g, and *ϕ* corresponds to the flax fiber mass fraction. Generally, the mathematical model suggested by Avrami [46] should be used to model the crystallization kinetics of flax/PLA composites. Avrami suggested a mathematical relation between the polymer’s relative crystallinity (χr) and the crystallization time (*t*). The Avrami equation does not apply to cases with primary and secondary crystallization. This equation only applies to the linear part of the Avrami plot [47,48]. Owing to the nonlinearity of relative crystallinity with time, however, a parallel Avrami model that describes both primary and secondary crystallization was considered in this study [46]. Equation (2) describes the primary and secondary crystallization of the polymer and is generally used for PEEK [46]

(2) $$\chi_{r}\left(t\right)=w_{1}\left(1-\exp\left[-k_{1}t^{n_{1}}\right]\right)+w_{2}(1-\exp\left[-k_{2}t^{n_{2}}\right])$$

where *χ_r_* is the relative crystallinity of the polymer at time *t*, *w*_1_, and *w*_2_ are weight factors corresponding to primary and secondary crystallizations, respectively, *k*_*i*=1,2_ is the crystallization rate constant, and *n*_*i*=1,2_ is the Avrami exponent. The weight factors are defined as *w*_1_ + *w*_2_ = 1, indicating that in the absence of secondary crystallization, Equation (2) will transform into the conventional Avrami equation [46]. The coefficients in Equation (2) were identified using curve fitting in Python, Version 3.11.

The Avrami equation is applicable for isothermal conditions, and the equation in its current form cannot be used for nonisothermal conditions. Generally, in order to reduce the processing time, the isothermal steps are avoided. Hence, the crystallization occurs during the nonisothermal step, i.e., the cooling step of the process cycle. In order to analyze the nonisothermal crystallization kinetics, Ozawa [49] suggested a model for the nonisothermal step by dividing it into infinitesimally small isothermal steps. This was further modified by Liu et al. [49] by combining Ozawa and Avrami equations, (a.k.a. Mo’s model) and is written as follows.

(3) $$\log\beta =\frac{1}{m}\log\left(\frac{K\left(T\right)}{Z}\right)-\frac{n}{m}logt$$

(4) logβ=log ⁡FT−alog(T)

where *β* is the cooling rate, *n* and *m* are Avrami and Ozawa coefficients, respectively, *K*(*T*) is the crystallinity rate, *Z* is the rate constant considering both nucleation and growth. Equation (3) can be further simplified into Equation (4), where *F*(*T*) refers to the value of the cooling rate at a unit crystallization time at a given degree of crystallinity.

#### 2.5.2. Thermogravimetry (TGA) Analysis

To study the thermal stability of the flax/PLA composites, thermogravimetry analysis (TGA) was performed using a Mettler Toledo TGA/DSC1, Viroflay, France. The test samples had a mass between 6 and 9 mg. The samples were placed in a ceramic crucible and were heated at a constant rate of 10 °C/min from 25 °C to 500 °C under an inert atmosphere. At least three samples were tested for each composition to report the average values.

### 2.6. Mechanical Tests

#### 2.6.1. Flexural Test

The three-point flexural tests were performed at room temperature in a Zwick/Roell Z030 Universal Testing Mleaachine (ZwickRoell ltd., Hertfordshire, UK) fitted with a 30 kN static load cell. The flexural properties (flexural strength and modulus) of compression-molded materials were retrieved according to sample dimensions and support span set respecting the British standard BS EN ISO 178:2003 [50]. Samples were loaded under displacement control at 5 mm/min. Flexural strength and moduli were evaluated according to Equations (5) and (6).

(5) $$E=\frac{L^{3}m}{4bd^{3}}$$

(6) $$\sigma =\frac{3FL}{2bd^{2}}$$

where *L* is the length of the specimen, *b* and *d* are the width and thickness of the specimen, respectively, *m* is the ratio between the change in force *F* and the change in displacement *s*.

#### 2.6.2. Impact Test

The impact characteristics of composite samples were determined with a Charpy impact pendulum device type 5102 (Zwick GmbH, Ulm, Germany) according to ISO 179-2:2020 [51], at a room temperature of 21 °C. Flatwise positioning of the compression-molded un-notched samples was adopted, and a strike energy of 1 Joule was used to test five specimens (80 mm × 10 mm) for each composite type at a bearing distance of 48 mm.

## 3. Results and Discussion

### 3.1. Properties of Flax/PLA Non-Wovens

**Figure 3:**
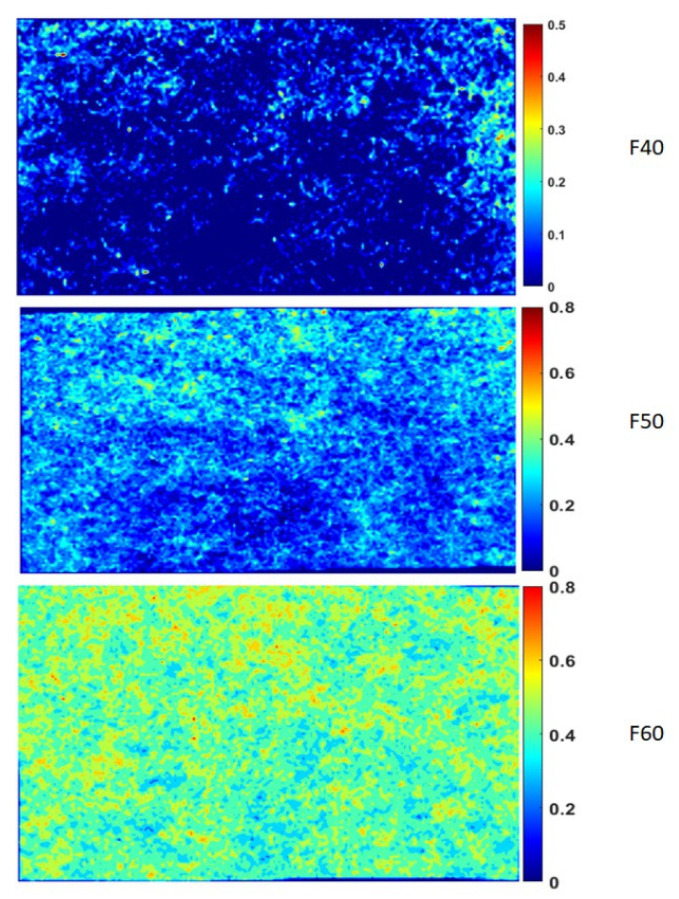
Contour plots of fiber distributions in different flax/PLA non-wovens.

This section presents the properties of non-wovens, such as the areal weight, thickness, and tensile strength (see Table 1). As mentioned in Section 2.1, the target areal weight of the fabrics has been set to 175 g/m^2^. However, deviations occurred due to the intrinsic variability of the carding process, which can lead to non-uniform distribution of flax and PLA fibers. Such variability is particularly distinguished in non-woven systems, where the fiber orientation and dispersion directly influence the mechanical performance of the composite, which is a phenomenon previously documented in the literature [15,32,40,52]. These variations can be caused by the non-uniformity of flax, PLA, or both fibers. Using the methodology described in Section 2.2, an initial trial with unconsolidated preform was made, but the distribution of fibers in the thickness direction made it difficult to quantify the variations. In Figure 3, the distribution of flax fibers is presented. The contour plot represents the normalized fiber distribution of the flax fibers within the preform. In the case of F40, the fibers are not well distributed, thus resulting in matrix-rich zones (indicated by dark blue zones), whereas F60 has rather uniform distributions with relatively low matrix-rich zones. These differences in fiber distribution can significantly affect the local impregnation and the subsequent change of mechanical properties [40,53]. Owing to these fiber clusters, the local rigidity of the preform during compaction can increase, leading to low compaction ratios. This local rigidity can be demonstrated by the void volume fraction variations within the composite plate, which will be discussed in Section 3.2.1.

#### 3.1.1. Crystallization Kinetics

In order to analyze the crystallization kinetics of flax/PLA composites, as mentioned in Section 2.5.1, Avrami and Mo’s equations were used. For the Avrami model, the relative crystallinity of the material was calculated using Equation (7)

(7) $${Χ}_{r}\left(t\right)=\frac{\int_{0}^{t}Q\left(t\right)dt}{\int_{0}^{t_{\infty }}Q\left(t\right)dt}$$

where *Q*(*t*) is the heat flow at time *t* and *t*_∞_ is the total isothermal duration. The relative crystallinity was then used to analyze the different crystallization kinetics of flax/PLA composites.

Table 2 shows the fitting parameters for the parallel Avrami equation, as presented in Section 2.5.2. It can be noticed that the crystallization of PLA occurs in two stages. The primary crystallization refers to the linear part of the curve, which is induced by the growth of crystal lamellas from nucleation sites. In contrast, secondary crystallization is linked to the completion of spherulite formation [54]. The linear part of the Avrami plot can be directly linked to the accelerated initial crystal growth. In works related to the study of PEEK crystallization, it was suggested to use the linear part of the curve to identify the Avrami exponent (*n*_1_ and *n*_2_) and use them to identify the other unknowns in Equation (2). In this study, we adopted a universal approach, where the model was fit to the whole curve without any presumptions concerning the Avrami exponents. Generally, the growth and number of crystals are governed by the primary crystallization. The molecular arrangement is more significant at this phase than at the secondary crystallization phase. The crystals would gradually grow in local zones until the maximum extent is reached, and the completion of this crystallization can be classified as secondary crystallization [55]. In Equation (2), the Avrami exponent *n* provides information concerning the nature of the nucleation and growth process of the crystals during the cooling phase. It is generally accepted that if the *n* value is close to 3, the growth of crystal structures is three dimensional, whereas if the value is between 2 and 3, the growth is two dimensional, such as circular lamellas. In Table 2, the *n*_1_ values indicate that the initial growth of crystals was two dimensional. Since most nucleating sites are on the surface of flax fibers, crystals tend to form 2D structures quickly. The *n*_2_ values indicate that the nucleation is sporadic in nature, and the crystals grow into 2D or 1D lamellar aggregates. The addition of flax fibers to PLA significantly improved the crystallization kinetics; however, no further improvement in crystallinity can be noticed. It has been previously reported in the literature that a threshold of flax content exists after which no significant improvement in crystallinity can be observed [56]. In this study, we can notice a similar phenomenon where the crystallization kinetics seem to be the same for all three flax/PLA non-wovens. The study of the growth rate of spherulites or crystal lamellas can be of interest since they can affect the local mechanical properties as well as the microstructure of the materials [32,54].

**Figure 4:**
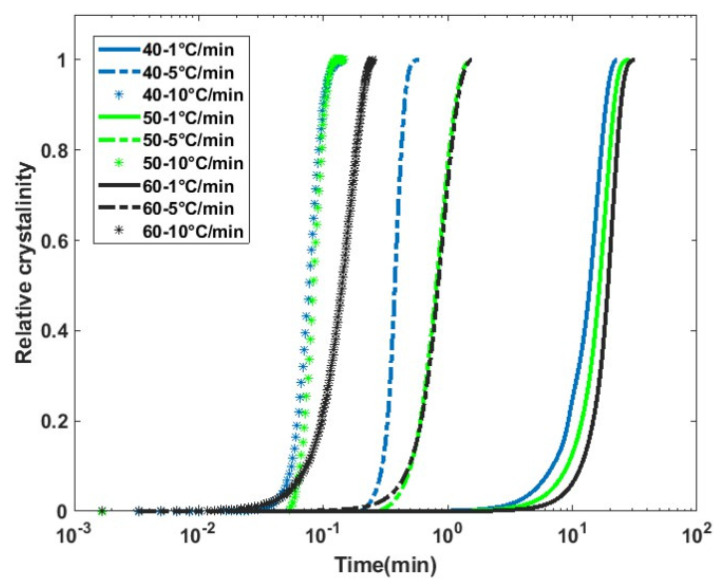
Nonisothermal crystallization kinetics of flax/PLA non-woven composites.

Nonisothermal crystallization kinetics (Figure 4) were modeled using the approach of Mo’s model. This model has been identified to be more accurate than conventional Avrami and Ozawa models in representing secondary crystallization kinetics (see Equation (4)). *F*(*T*) is the cooling rate parameter, which can be physically described as the cooling rate required to achieve a relative crystallinity at unit crystallization time. In other words, a higher value of *F*(*T*) indicates that a slower cooling rate is needed to achieve crystallization. In Table 3, the coefficients of Equation (4) are presented. The *F*(*T*) values for the composites are much smaller than those of the pure PLA matrix, indicating that the crystallization process is faster for a given cooling rate. This is expected since fibers act as nucleating sites and increase the crystallization rate, as observed in the case of isothermal crystallization.

#### 3.1.2. Thermal Degradation Analysis

**Figure 5:**
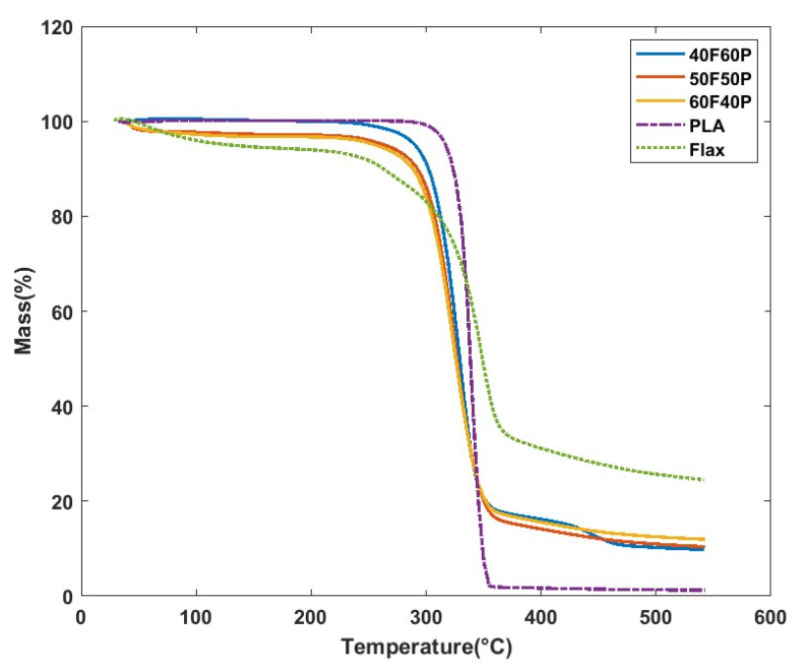
Thermal behavior of flax/PLA composites.

Figure 5 illustrates the thermograms from thermogravimetric analysis (TGA) of various flax/PLA composites, including pure flax and pure PLA for comparative analysis. These thermograms reveal distinct thermal degradation patterns, showing that the majority of mass loss occurs around 300 °C for all specimens, indicative of thermal decomposition stages for both the flax and PLA components. Initial degradation begins at approximately 220 °C, aligning with the known thermal instability of flax fibers at elevated temperatures [7]. As flax fiber mass fraction increases, the thermal stability of the composites improves slightly, attributed to the higher residual char content, which acts as a thermal barrier, slowing down further decomposition. The initial mass reduction below 100 °C is likely due to the evaporation of inherent moisture within the specimens, which is a common characteristic in natural fibers. The mass loss observed between 100 °C and 200 °C can be linked to the decomposition of organic compounds such as pectin, lignin, and waxes within the flax fibers, contributing to a gradual weight decrease in the flax and its composites [57]. This breakdown of organic matter occurs prior to the onset of main degradation, which involves the degradation of the PLA matrix and further breakdown of cellulose. Interestingly, the thermal responses of F50 and F60 composites are nearly identical, implying two possible scenarios: a non-uniform distribution of flax and PLA in the preform or a threshold beyond which additional flax integration no longer improves the thermal stability significantly. Higher flax fiber mass fraction may provide enhanced stability by increasing the amount of char, which acts as an insulating layer, slowing the rate of thermal degradation in the composite. Additionally, the increased char residue at high flax mass fractions could imply a protective effect, further enhancing the thermal stability and integrity of the composite materials during prolonged thermal exposure. These findings emphasize the role of flax mass fraction in improving composite performance under heat, which is crucial for applications where thermal endurance is required at elevated temperatures.

### 3.2. Mechanical Properties of Flax/PLA Composites

#### 3.2.1. Void Volume Fraction and Crystallinity of Composites

**Figure 6:**
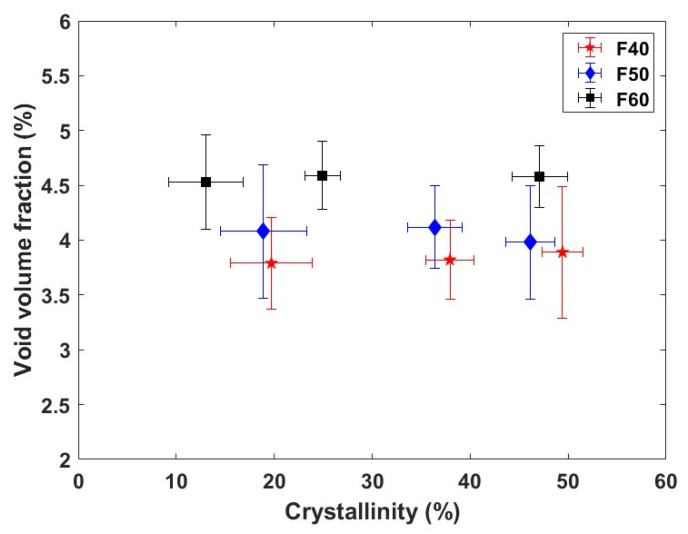
Residual void content and crystallinity of flax/PLA composites.

In Figure 6, the void volume fraction and crystallinity of flax/PLA composites are presented, highlighting critical structural characteristics influenced by the flax fiber mass fraction within the preforms. As hypothesized in Section 3.1, the void volume fraction increases as the flax fiber mass fraction increases. This rise in void volume fraction can be attributed to two primary factors: the spatial distribution of flax fibers and the mechanical behavior of the preform under compaction. Firstly, the non-uniform distribution of fibers within the composite can lead to local agglomerations of flax fibers, forming a more tortuous path for the PLA melt to penetrate and fully impregnate the reinforcement. This phenomenon not only hinders uniform PLA distribution but also creates micro-regions within the composite where resin infiltration is incomplete, leading to air entrapment. Secondly, the accumulation of fiber clusters increases the local rigidity of the preform, which restricts compaction and can result in uneven density across the composite. This variability in compaction can further contribute to the development of discrete compaction zones, with varying degrees of fiber packing and air retention. The void volume fraction in these composites can be classified into two primary categories: air-entrapment voids and fiber–matrix interfacial voids. Air-entrapment voids are primarily due to the incomplete displacement of air pockets during the impregnation phase. Meanwhile, voids at the fiber–matrix interface arise due to insufficient bonding between flax fibers and PLA. These interfacial voids can significantly influence the composite’s mechanical properties, as they weaken the stress transfer between the fibers and the polymer matrix, reducing the overall structural integrity [58]. Additionally, the degree of crystallinity of PLA is sensitive to flax fiber mass fraction and distribution. A higher flax fiber mass fraction can increase the number of nucleation sites for the crystallization of the PLA matrix, potentially enhancing its thermal stability and stiffness. An excess of voids due to poor impregnation and interfacial gaps may disrupt the crystallinity, however, as air pockets interrupt the molecular alignment needed for uniform crystallite formation.

#### 3.2.2. Flexural Properties

**Figure 7:**
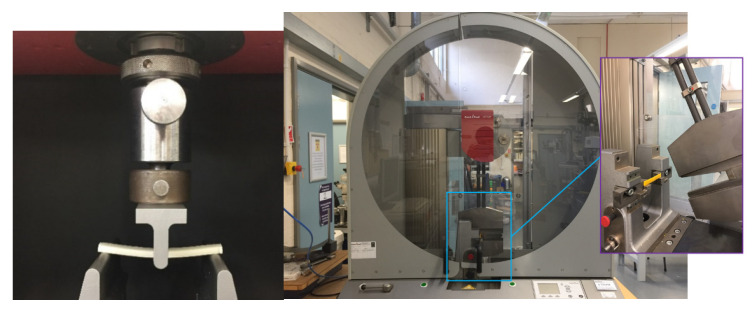
Experimental setups for mechanical characterization: flexural tests (**left**) and impact tests (**right**).

**Figure 8:**
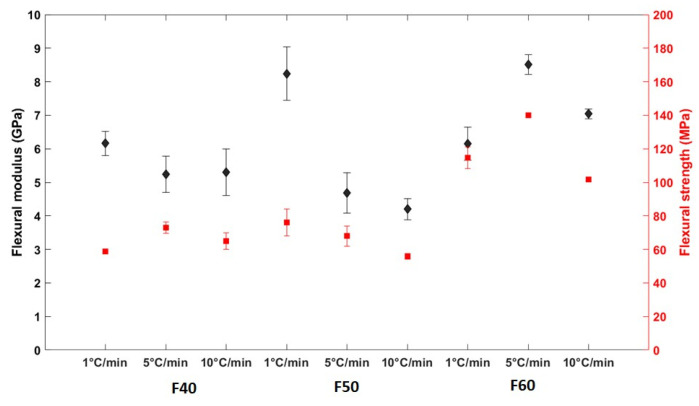
Flexural properties of flax/PLA composites subjected to different cooling rates.

In this study, flexural tests were carried out using a three-point bending method as shown in Figure 7. The influence of cooling rate on the flexural properties of flax/PLA composites can be observed in Figure 8. F40 and F50 composites exhibited a trend that was well reported in the literature [34] (see Figure 8). With an increase in the cooling rates, the F40 and F50 composites showed a drop in flexural modulus, which is coherent with the cooling rate and degree of crystallinity. Conversely, the trend for the other F60 flax/PLA composites was different. This can be caused by the lack of impregnation and increased void volume fraction, as stated in Section 2.2. It can thus be inferred that the distribution of PLA and flax fibers in the non-woven fabric was non-uniform, leading to fiber-rich and matrix zones. However, it has been observed that the flexural moduli obtained in the current study are slightly higher than the values reported in the literature [59,60].

It is difficult to find a global trend in the flexural strength against the cooling rate. We can also see that the flexural strength of F60 is significantly greater than that of F40. It can be hypothesized that this difference can be linked to the flax fiber mass fraction. With the increase in the flax fiber mass fraction, the reinforcing effect of flax fibers becomes greater. At the same time, the probability of available nucleating zones increases significantly, enabling easier crystallization of PLA and resulting in a higher degree of crystallization. For F60 composites, the flexural strength increases with a decrease in the cooling rate (from 10 to 5 °C/min), indicating the transition of the properties of composites into a relatively brittle behavior. A similar trend can also be noticed for F50 composites. In the case of F40 composites, a drop in the flexural strength was noticed with a decrease in the crystallinity.

To evaluate the performance of flax/PLA composites, their specific flexural modulus and strength are compared with those of commercial short glass fiber thermoplastic composites designed for semi-structural applications [61]. In our work, flax/PLA composites with a 40% fiber mass fraction exhibit average specific flexural modulus and strength of 4.11 GPa/g/cm^3^ and 55 MPa/g/cm^3^, respectively. These values are comparable to the specific flexural modulus and strength of short glass fiber composites (30% fiber mass volume fraction), which are 3.5 GPa/g/cm^3^ and 100 MPa/g/cm^3^, respectively. Although the flexural strength of flax/PLA composites is lower, it is expected that increasing the flax fiber mass fraction can reduce this difference. When F60 composites are analyzed, they demonstrate superior average specific flexural modulus and strength of 6.03 GPa/g/cm^3^ and 100.45 MPa/g/cm^3^, respectively. This implies that composites manufactured under optimal process conditions and material configurations can offer a promising alternative to short glass fiber composites for such applications.

#### 3.2.3. Impact Properties

**Figure 9:**
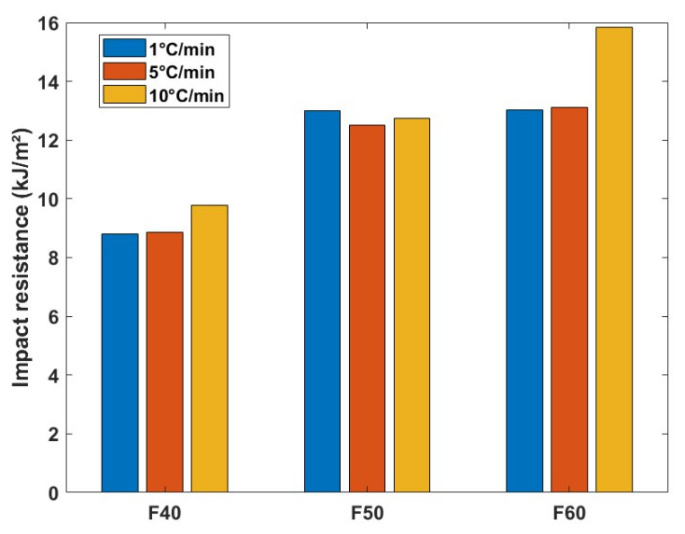
Impact properties of flax/PLA composites subjected to different cooling rates.

In Figure 9, the results of the impact resistance of flax/PLA composites under varying cooling rates reveal significant differences, highlighting the influence of the cooling rate on the material brittleness. As the cooling rate decreases and crystallinity increases, the composites exhibit a more brittle behavior, leading to reduced impact resistance, which is a phenomenon well reported in the literature [9,62]. Specifically, the impact resistance of F40 composites decreased by approximately 11% under a slow cooling rate of 1 °C/min, while F60 composites exhibited a more pronounced reduction of 18% at the same cooling rate. Interestingly, F50 composites showed minimal variation in impact resistance across different cooling rates, implying that the fiber and matrix distribution may play a stabilizing role in this configuration.

Bax and Mussig reported 11.13 kJ/m^2^ impact strength for random flax/PLA composites with 30% of fiber mass fraction, whereas Foruzanmehr et al. [63] reported an impact strength of 18 kJ/m^2^ for UD flax/PLA composites with 34% of fiber mass fraction. Compared to the results in the literature [64], the current composites exhibit better impact properties, and the distribution of flax fibers is likely to have a substantial influence on the composite’s toughness. Higher flax mass fraction can increase the formation of fiber-rich zones, which may lead to uneven crystallization and localized brittleness, reducing impact performance. Furthermore, the differences in void volume fraction have a critical influence on the impact resistance. Composites with high void volume fraction are less capable of withstanding impact loads due to insufficient energy absorption and accelerated crack propagation. The voids, especially those at the fiber–matrix interface, act as stress concentrators, which weaken the material and lead to faster fracture under impact [10]. Thus, the cooling rate and fiber distribution both play crucial roles in determining the impact properties of flax/PLA composites. A slower cooling rate generally promotes higher crystallinity, which can compromise impact resistance by increasing brittleness, even if it is beneficial to improve the stiffness.

## 4. Conclusions

This study systematically investigated the crystallization kinetics and mechanical properties of flax/PLA non-woven composites under different thermal processing conditions. The composites were fabricated using a dry layup and needle-punching approach, achieving flax fiber mass fractions up to 60%. The results showed that a 40% flax fiber mass fraction led to a 25% improvement in flexural modulus, while a 50% flax fiber mass fraction increased it by 100% under slow cooling conditions. Impact properties, however, decreased by approximately 11% for composites subjected to a cooling rate of 1 °C/min, implying increased brittleness. Void content analysis indicated that higher fiber loading led to localized matrix-rich and fiber-rich zones, influencing structural uniformity. Isothermal crystallization kinetics were accurately modeled using a parallel Avrami approach, revealing non-linear growth behaviors, while non-isothermal kinetics demonstrated significantly enhanced crystallization rates due to the nucleating effect of flax fibers, with F (T) values for flax/PLA composites that are 20–25% lower than those of pure PLA. The specific mechanical properties were excellent, with flexural modulus and strength reaching up to 6.03 GPa/g/cm^3^ and 100.45 MPa/g/cm^3^, respectively, comparable to those of short glass fiber composites with a 30% fiber mass fraction. These results suggest that flax/PLA composites are promising for semi-structural applications, providing a sustainable alternative with enhanced lightweight performance. Furthermore, in addition to their improved mechanical performance, it would be interesting to carry out a detailed LCA analysis to understand the influence of process parameters on the environmental indicators.

## Figures and Tables

**Table 1:** Properties of non-wovens.

Non-Woven	Areal Weight (g/m^2^)	Thickness (mm)	Strength (N/50 mm)		Elongation (%)	
			MD	CD	MD	CD
F40	161.67 ± 4.19	1.80 ± 0.045	64.0	117.9	73.0	43.5
F50	183.16 ± 0.86	1.88 ± 0.01	52.0	85.9	70.7	43.1
F60	171.67 ± 1.24	1.78 ± 0.02	41.2	63.9	72.1	44.3

**Table 2:** Parameters for isothermal crystallization model.

Non-Woven	Temperature (°C)	w_1_	k_1_ (10^−7^ s^−1^)	n_1_	w_2_	k_2_(10^−7^ s^−1^)	n_2_
F40	80	0.65	1.083	2.182	0.344	2.179	1.369
	100	0.63	1.285	2.311	0.363	2.415	1.457
	120	0.62	1.251	2.314	0.372	3.343	1.413
F50	80	0.64	0.961	2.19	0.353	2.496	1.346
	100	0.62	1.284	2.307	0.371	3.071	1.414
	120	0.62	1.253	2.311	0.376	3.975	1.382
F60	80	0.64	0.965	2.195	0.353	2.336	1.357
	100	0.62	1.272	2.311	0.371	2.702	1.437
	120	0.62	1.264	2.312	0.378	3.801	1.389

**Table 3:** Coefficients of Mo’s model.

	F40		F50		F60		PLA	
χ (%)	F (T)	a	F (T)	a	F (T)	a	F (T)	a
20	1.042	0.46	0.9328	0.3723	1.01	0.3618	1.409	0.4873
40	1.138	0.448	1.289	0.444	1.395	0.4704	1.526	0.4794
60	1.193	0.4419	1.348	0.44	1.47	0.4969	1.606	0.4765
80	1.242	0.4395	1.393	0.4303	1.54	0.4835	1.681	0.4814

## Data Availability

Data is contained within the article. The original contributions presented in this study are included in the article. Further inquiries can be directed to the corresponding author(s).
